# Genetic Analyses of Heme Oxygenase 1 (*HMOX1*) in Different Forms of Pancreatitis

**DOI:** 10.1371/journal.pone.0037981

**Published:** 2012-05-30

**Authors:** Sebastian Weis, Moritz Jesinghaus, Peter Kovacs, Dorit Schleinitz, Robert Schober, Claudia Ruffert, Max Herms, Henning Wittenburg, Michael Stumvoll, Matthias Blüher, Robert Grützmann, Hans-Ulrich Schulz, Volker Keim, Joachim Mössner, Peter Bugert, Heiko Witt, Joost P. H. Drenth, Knut Krohn, Jonas Rosendahl

**Affiliations:** 1 Department of Internal Medicine, Neurology and Dermatology, Division of Gastroenterology and Rheumatology, University of Leipzig, Leipzig, Germany; 2 Department of Internal Medicine, Neurology and Dermatology, Division of Endocrinology, University of Leipzig, Leipzig, Germany; 3 IFB Adiposity Diseases, University of Leipzig, Leipzig, Germany; 4 Department of General, Thoracic and Vascular Surgery, University Hospital Carl Gustav Carus, Technische Universität Dresden, Dresden, Germany; 5 Department of Surgery, Otto-von-Guericke University Magdeburg, Magdeburg, Germany; 6 Institute of Transfusion Medicine and Immunology, Medical Faculty Mannheim, Heidelberg University, German Red Cross Blood Service of Baden-Württemberg-Hessen, Mannheim, Germany; 7 Department of Pediatrics, Else Kröner-Fresenius-Zentrum für Ernährungsmedizin (EKFZ) & Zentralinstitut für Ernährungs- und Lebensmittelforschung (ZIEL); Technische Universität München, Munich, Germany; 8 Department of Gastroenterology and Hepatology, Radboud University Nijmegen Medical Centre, Nijmegen, The Netherlands; 9 Interdisciplinary Centre for Clinical Research Leipzig, Core-Unit DNA Technologies, University of Leipzig, Leipzig, Germany; Innsbruck Medical University, Austria

## Abstract

**Background:**

Heme oxygenase 1 (HMOX1) is the rate limiting enzyme in heme degradation and a key regulator of inflammatory processes. In animal models the course of pancreatitis was ameliorated by up-regulation of HMOX1 expression. Additionally, carbon monoxide released during heme breakdown inhibited proliferation of pancreatic stellate cells and might thereby prevent the development of chronic pancreatitis (CP). Transcription of HMOX1 in humans is influenced by a *GT*-repeat located in the promoter. As such, *HMOX1* variants might be of importance in the pathogenesis of pancreatitis.

**Methods:**

The *GT*-repeat and SNP rs2071746 were investigated with fluorescence labelled primers and by melting curve analysis in 285 patients with acute pancreatitis, 208 patients with alcoholic CP, 207 patients with idiopathic/hereditary CP, 147 patients with alcoholic liver cirrhosis, and in 289 controls, respectively. *GT*-repeat analysis was extended to a total of 446 alcoholic CP patients. In addition, we performed DNA sequencing in 145 patients with alcoholic CP, 138 patients with idiopathic/hereditary CP, 147 patients with alcoholic liver cirrhosis, and 151 controls. Exon 3 screening was extended to additional patients and controls.

**Results:**

S- and L-alleles of the *GT*-repeat, genotypes and alleles of SNP rs2071746 and non-synonymous variants detected by sequencing were found with similar frequencies in all groups.

**Conclusions:**

Although functional data implicate a potential influence of *HMOX1* variants on the pathogenesis of pancreatitis, we did not find any association. As rare non-synonymous *HMOX1* variants were found in patients and controls, it is rather unlikely that they will have functional consequences essential for pancreatitis development.

## Introduction

Acute pancreatitis (AP) is a potentially life threatening disease presenting with a wide clinical spectrum that ranges from mild discomfort to multi organ failure [1]. In the Western world, the leading causes of AP are chole(docho)lithiasis and alcohol abuse. Apart from the p.N34S *SPINK1* variant, no other genetic association with AP has been confirmed so far [2]. Chronic pancreatitis (CP) is a relapsing inflammatory disease resulting in a permanent impairment of exocrine and endocrine organ function in many cases. In industrialised countries, chronic alcohol abuse is the major underlying cause while nicotine abuse is an important contributing factor [3]. Although several genetic associations have been described for idiopathic and hereditary CP (ICP, HP) little is known about genetic alterations that contribute to the pathogenesis of AP and alcoholic CP (ACP) [4]–[10].

There are 2 functional heme oxygenase isoforms, the inducible HMOX1 (also designated as HO-1; OMIM*141250) and the constitutively expressed HMOX2 [11]. HMOX1 is a key regulator of inflammatory processes as moderate over-expression of HMOX1 protects cells, whereas excessive HMOX1 expression is harmful [12]. Cellular HMOX1 content is transcriptionally regulated and gene expression is induced by many different stimuli such as heavy metals, inflammation, UV radiation, oxidative stress and even by HMOX1 itself after translocalisation to the nucleus [13]. Gene transcription in humans is modulated by the length of a dinucleotide *GT*-repeat in the promoter classified in short alleles and long alleles [14]. Previous studies demonstrated an inducible elevation of HMOX1 activity in presence of S-alleles. For example HMOX1 mRNA content and oxidative stress induced HMOX1 enzymatic activity are significantly higher in S-allele carriers [15].

In animal models of experimental pancreatitis, expression of HMOX1 is up-regulated in pancreatic islet and acinar cells and HMOX1 as well as carbon monoxide (CO) act protective [16]–[18]. Moreover, HMOX1 induction improves outcome after pancreas transplantation and ameliorates microcirculatory derangements after ischemia and reperfusion [19], [20]. For the development of CP, fibrotic remodelling of the pancreatic parenchyma is an essential step, with a crucial role for pancreatic stellate cells (PSC) [21]. Of note, PSC proliferation is inhibited by CO and endogenous CO is mainly produced during the breakdown of heme by microsomal HMOXs [22].

As such, it is reasonable to portend that different *GT*-repeat alleles or other *HMOX1* genetic alterations contribute to the pathogenesis of different pancreatitis phenotypes. To investigate the role of *HMOX1* alterations in pancreatitis, we extensively screened the *GT*-repeat, SNP rs2071746, and the coding sequence in up to 446 patients with different forms of pancreatitis, 147 patients with alcoholic liver cirrhosis (ALC) and up to 413 healthy controls.

## Materials and Methods

### Patients and Controls

The study was approved by the medical ethical review committee of the University of Leipzig, Germany (Approval: 376-11-12122011). All patients gave written informed consent. AP was diagnosed and categorised according to the Atlanta classification [1]. We categorised patients into a group with a mild disease course (only local complications) and a group with severe disease course (additionally systemic complications). Diagnosis of CP was based on two or more of the following findings: Presence of a history of recurrent pancreatitis or recurrent abdominal pain typical for CP, pancreatic calcifications and/or pancreatic ductal irregularities revealed by endoscopic retrograde pancreaticography or by magnetic resonance imaging of the pancreas and/or pathological sonographic findings. ACP was defined in patients who had consumed more than 80 g/d alcohol for at least two years in men and more than 60 g/d for women [10]. HP was diagnosed when one first-degree relative or two or more second-degree relatives suffered from recurrent AP or CP without any apparent precipitating factor. ICP was diagnosed in the absence of a positive family history or known precipitating factors.

ALC was diagnosed according to results of liver biopsy (fibrosis stage 4) or due to unequivocal clinical and laboratory findings in men who consumed more than 80 g/d and in women who consumed more than 60 g/d for at least 10 years. Such findings were abnormal levels of aminotransferases, gamma glutamyl-transpeptidase, coagulation tests, serum albumin concentration, platelet count, complications related to liver cirrhosis like oesophageal varices, ascites, hepatic encephalopathy and typical liver morphology in ultra-sound or computed tomography. Other aetiologies of liver cirrhosis were excluded by standard laboratory tests.

In total, we analysed the *GT*-repeat and SNP rs2071746 in 285 patients with AP (176 male, age range 9–89 years, median 53 years; aetiology: biliary = 115, alcoholic = 68, idiopathic = 66, post-operative = 15, post-ERCP = 8, traumatic = 6, hyperlipoproteinaemia = 5, hyperparathyroidism = 1, drugs = 1; disease course: mild = 205, severe = 80), 208 ACP (199 male, age range 21–79 years, median 47 years), 207 ICH/HP (100 male, age range 3–77 years, median 29 years), 147 ALC patients (111 male, age range 32–79 years, median 56 years), and in 289 controls (88 male, age range 20–81 years, median 47 years). In addition, we investigated the *GT*-Repeat in 238 ACP patients (210 male, age range 23–75 years, median 47 years).

In 145 German patients with ACP (124 male, age range 21–69 years, median 45 years), 138 patients with ICP/HP (60 male, age range 3–75 years, median 33 years), 147 patients with ALC (see above) and in 151 controls (50 male, age range 20–70 years, median 45.5 years) all coding regions and transitions to non-coding regions were analysed by uni-directional DNA sequencing. We screened *HMOX1* exon 3 in additional 301 ACP patients (267 male, age range 23–73 years, median 44 years), 110 ICP/HP patients (41 male, age range 3–75 years, median 32.5 years), and in 262 controls (175 male, age range 36–98 years, median 59 years) by direct DNA sequencing. The controls investigated with the different methods were blood donors from South-West and East Germany.

### Genotyping

#### Analysis of the GT-repeat

Primers were synthesised according to the published nucleotide sequences (*HMOX1*: GenBank: NM_002133.2 and NG_023030.1). For classification of the *GT*-repeat located in the *HMOX1* promoter, we performed PCR with fluorescent labelled primers under the conditions described below and 35 cycles with an annealing temperature of 58°C: Forward primer 5′-FAM-AGAGCCTGCAGCTTCTCAGA-3′, reverse Primer 5′-TGGAGAGGAGCAGTCATATG-3′. We loaded PCR products together with a size standard onto an ABI 3100 fluorescence sequencer (Applied Biosystems) for fragment analysis and determined the length of the amplified PCR product as the number of *GT*-repeats. We classified the products containing *GT*-repeats according to the literature in short repeats (S<25) and long repeats (L≥25) [23].

### Melting curve analysis of rs2071746

We performed PCR in the LightCycler 480 instrument (Roche Diagnostics) under the following conditions (volumes see below): initial denaturation at 95°C for 5 minutes followed by 45 cycles with denaturation at 95°C for 5 seconds, annealing at 55°C for 20 seconds, primer extension at 72°C for 20 seconds. Primers had the following oligonucleotide sequences: Forward primer 5′-CAAGCAGTCAGCAGAGGATTC-3′, reverse primer 5′-GCAGGCTCTGGGTGTGATT-3′. We performed melting curve analysis using a pair of fluorescent resonance energy transfer (FRET) probes. FRET probes were designed complementary to the mutated sequence. Probes were devised and synthesised by TIB Molbiol (Berlin, Germany): Anchor probe 5′-LC640-GCGCTGCCTCCCAGCTTTCTGGAA-3′, sensor probe 5′-CACCAGGCTATTGCTCTGAGC-FL-3′. Analytical melting included the following steps: 95°C for 5 seconds, 40°C for 20 seconds and an increase to 80°C at a 0.29°C/s ramp rate.

### Polymerase Chain Reaction and DNA sequencing

We extracted genomic DNA from peripheral blood leukocytes and performed PCR using 0.75 U AmpliTaq Gold polymerase (Applied Biosystems), 400 µM dNTPs, 1.5 mM MgCl_2_ and 0.1 µM of each primer in a total volume of 25 µl. Cycle conditions were as follows: an initial denaturation for 12 minutes at 95°C followed by 48 cycles of 20 seconds denaturation at 95°C, 40 seconds annealing at specific temperatures, 90 seconds primer extension at 72°C and a final extension for 2 minutes at 72°C in an automated thermal cycler. Oligonucleotide sequences and annealing temperatures of the primers are listed in Table S1.

We digested PCR products with shrimp alkaline phosphatase (USB) and exonuclease I (GE Healthcare) and performed cycle sequencing using BigDye terminator mix (Applied Biosystems). We purified reaction products with ethanol precipitation and loaded them onto an ABI 3100 fluorescence sequencer (Applied Biosystems). Mutations are described according to the nomenclature recommended by the Human Genome Variation Society (http://www.hgvs.org/mutnomen) following a common consensus with mutation numbering which defines the A of the ATG start codon as nucleotide +1.

### Statistics

We tested the significance of the differences between variant frequencies in affected individuals and controls by two-tailed Fisher's Exact test. *P*-values were calculated using GraphPad Prism (v 4.03). For SNPs we utilised a dominant model, defined as AA vs. AG+GG (e.g. for c.736+226A>G, rs2269533), for calculations and considered *p*-values <0.05 to be of statistical significance. In addition, calculations were performed following a recessive model (AA+AG vs. GG) and for allele frequencies. We used the first allele in the variant description as the major allele (example above for c.736+226A>G, rs2269533). The *p*-values are shown without correcting for multiple testing.

## Results

### Fragment length analyses of the promoter GT-repeat

Our analysis of the *GT*-repeat revealed repeats ranging from 12 to 40 *GT*s. In all groups we detected alleles with 23 (AP 19.7%, ACP 24.2%, ICP/HP 21.1%, ALC 22.8%, controls 19.7%) and 30 repeats (AP 42.5%, ACP 45.6%, ICP/HP 48.1%, ALC 41.5%, controls 43.8%) with highest frequencies in accordance with previous reports [23]. In AP patients, frequencies of S- and L-alleles were similar to frequencies obtained in controls (S-allele: 173/578, 29.9%) even after categorization in mild (S-allele: 134/410, 32.7%; *p*-value 0.4) and severe disease course (S-allele: 55/160, 34.4%; *p*-value 0.3) (mild vs. severe: *p*-value 0.7). In our first screen of 208 ACP patients, we found S-alleles (145/416, 34.9%) more common in patients than in controls (*p*-value 0.1). Although this finding was not statistically significant, we extended our analysis and after screening of additional 238 ACP patients we strengthened the preliminary results and ruled out an association (*p*-value 0.4). In all other groups distribution of alleles was similar in patients and controls (Table 1).

**Table 1:** *GT*-repeat analysis in acute pancreatitis (AP), alcoholic chronic pancreatitis (ACP), idiopathic/hereditary chronic pancreatitis (ICP/HP), and alcoholic liver cirrhosis (ALC) patients and in controls.

Alleles	Patients	Controls	*p*-Value
	**AP**		
S	189/570 (33.2%)	173/578 (29.9%)	n.s
L	381/570 (66.8%)	405/578 (70.1%)	
	**ACP**		
S	286/892 (32.1%)	173/578 (29.9%)	n.s
L	606/892 (67.9%	405/578 (70.1%)	
	**ICP/HP**		
S	125/412 (30.3%)	173/578 (29.9%)	n.s
L	287/412 (69.7%)	405/578 (70.1%)	
	**ALC**		
S	91/294 (31%)	173/578 (29.9%)	n.s
L	203/294 (69%)	405/578 (70.1%)	

S-alleles were defined as <25 *GT*-repeats, whereas L-alleles represented ≥25 *GT*-repeats. Abbreviations: S = S-allele, L = L-alleles, n.s. = not significant.

### SNP analyses

There was no difference in the genotype and allele frequency distribution of SNP rs2071746 (g.4613A>T) in AP, ACP, ICP/HP, and ALC patients compared to controls (Table 2). Even after categorization into mild (*A*-allele: 229/410, 55.9%; Genotype: AA 60/205, 29.3%; AT 109/205, 53.2%) and severe (*A*-allele: 84/160, 52.5%; Genotype: AA 25/80, 31.3%; AT 34/80, 42.5%) disease course no difference was obtained in the AP group compared to controls (all *p*-values not significant).

**Table 2:** Genotype data of SNP rs2071746 in acute pancreatitis (AP), alcoholic chronic pancreatitis (ACP), idiopathic/hereditary chronic pancreatitis (ICP/HP), and alcoholic liver cirrhosis (ALC) patients and in controls.

Variant	Genotype	Patients	Controls	*p*-Value
g.4613A>Trs2071746		**AP**		
	AA	85/285 (29.8%)	96/289 (33.2%)	n.s.
	AT	143/285 (50.2%)	142/289 (49.1%)	
	TT	57/285 (20%)	51/289 (17.6%)	
		**ACP**		
	AA	62/208 (29.8%)	96/289 (33.2%)	n.s.
	AT	107/208 (51.4%)	142/289 (49.1%)	
	TT	39/208 (18.8%)	51/289 (17.6%)	
		**ICP/HP**		
	AA	73/207 (35.3%)	96/289 (33.2%)	n.s.
	AT	104/207 (50.2%)	142/289 (49.1%)	
	TT	30/207 (14.5%)	51/289 (17.6%)	
		**ALC**		
	AA	51/147 (34.7%)	96/289 (33.2%)	n.s.
	AT	68/147 (46.3%)	142/289 (49.1%)	
	TT	28/147 (19%)	51/289 (17.6%)	

Abbreviations: n.s. = not significant.

### DNA sequencing

By DNA sequencing of the complete coding region, we found five common *HMOX1* variants (c.144+4T>C, rs17885925; c.145−19C>T, rs17879606; c.736+52delTinsTGGCTGTCTGACT, rs17882597; c.736+226A>G, rs2269533; c.736+270T>C, rs2269534) in patients and controls (Table S2). Apart from SNP rs2269534 in ACP patients, frequencies of all SNPs were similar in patients and controls and the distribution showed no statistical significant difference when allele frequencies and genotypes were compared. In ACP patients allele frequencies of SNP rs2269534 differed significantly between patients (*T*-allele: 166/260, 63.9%) and controls (195/270, 72.2%) (*p*-value 0.04, OR 1.5, 95% CI 1.02–2.13), but the association did not withstand Bonferroni correction (*p_c_*-value 0.2).

We identified non-synonymous variants and variants with a minor allele frequency <5% in 25/145 (17.2%) ACP patients and in 27/151 (17.9%) controls (Table 3). Non-synonymous variant c.19G>C (p.D7H, rs2071747) was detected in similar frequencies in ACP patients (14/145, 9.7%) and controls (15/151, 9.9%). We found variants c.379G>T (p.E127X) and c.577C>T (p.P193S) in patients only (1/145, 0.7%; 2/145, 1.4%), whereas we detected variant c.101T>C (p.M34T) in one control only (1/151, 0.7%). None of the variants showed a statistical significant difference of their frequencies in patients and controls.

**Table 3:** Rare variants identified by sequencing of *HMOX1* in alcoholic chronic pancreatitis (ACP), idiopathic/hereditary chronic pancreatitis (ICP/HP), and alcoholic liver cirrhosis (ALC) patients and controls and their location within *HMOX1*.

Variant	Location	ACP	ICP/HCP	ALC	Controls	*p*-Value
c.−120A>T	5′-UTR	0/145	0/138	0/147	1/151 (0.7%)	n.s.
c.−88T>C	5′-UTR	0/145	0/138	0/147	1/151 (0.7%)	n.s.
c.−13C>T (rs9282701)	5′-UTR	1/145 (0.7%)	0/138	1/147 (0.7%)	1/151 (0.7%)	n.s.
c.19G>C (rs2071747), p.D7H	Exon 1	14/145 (9.7%)	11/138 (7.2%)	11/147 (7.5%)	15/151 (9.9%)	n.s.
c.101T>C, p.M34T	Exon 1	0/145	0/138	0/147	1/151 (0.7%)	n.s.
c.23+28_29delCGGGACG	Intron 1	1/145 (0.7%)	1/138 (0.7%)	1/147 (0.7%)	0/151	n.s.
c.23+91G>A	Intron 1	1/145 (0.7%)	3/138 (2.2%)	0/147	2/151 (1.3%)	n.s.
c.23+241C>T	Intron 1	0/145	2/138 (1.5%)	2/147 (1.4%)	1/151 (0.7%)	n.s.
c.144+206delTGT	Intron 2	0/145	0/138	1/147 (0.7%)	1/151 (0.7%)	n.s.
c.144+246C>T	Intron 2	0/145	0/138	0/147	1/151 (0.7%)	n.s.
c.144+272C>T	Intron 2	0/145	0/138	1/147 (0.7%)	0/151	n.s.
c.234C>T, (p.( = ))	Exon 3	0/145	0/138	0/147	1/151 (0.7%)	n.s.
c.330C>T, (p.( = ))	Exon 3	1/145 (0.7%)	0/138	0/147	1/151 (0.7%)	n.s.
c.379G>T, p.E127X	Exon 3	1/145 (0.7%)	0/138	0/147	0/151	n.s.
c.407G>A, p.R136H	Exon 3	0/145	1/138 (0.7%)	0/147	0/151	n.s.
c.577C>T, p.P193S	Exon 3	2/145 (1.4%)	0/138	0/147	0/151	n.s.
c.621C>T, (p.( = ))	Exon 3	1/145 (0.7%)	0/138	0/147	0/151	n.s.
c.736+322G>A	Intron 4	0/145	1/138 (0.7%)	0/147	0/151	n.s.
c.736+331G>A	Intron 4	0/145	0/138	0/147	1/151 (0.7%)	n.s.
c.*71C>G	3′-UTR	0/145	2/138 (1.5%)	0/147	0/151	n.s.
c.*149A>G	3′-UTR	3/145 (2.1%)	1/138 (0.7%)	1/147 (0.7%)	0/151	n.s.

Abbreviations: n.s. = not significant.

In the cohort of ICP/HP patients, only one patient (1/138, 0.7%) carried a non-synonymous variant (c.407G>A, p.R136H) apart from c.19G>C (11/138, 7.2%). In patients with ALC, no patient carried a non-synonymous variant other than c.19G>C (11/147, 7.5%). Overall, we observed no significant difference in the distribution of variants in both patient groups compared to controls.

To elucidate the role of non-synonymous exon 3 variants (c.379G>T, c.407G>A, and c. 577C>T), we extended our analyses (Table 4). Subsequently, we found variant c.379G>T in 1/446 (0.2%) ACP patients, but not in ICP/HP patients or controls, variant c.577C>T in 2/446 (0.4%) ACP patients and in 2/248 (0.8%) ICP/HP patients compared to 2/413 (0.5%) controls, and variant c.407G>A in 1/248 (0.4%) ICP/HP patients, but not in 413 controls. In addition, we identified a hitherto undescribed variant c.473C>T (p.P158L) in one control subject. In summary, none of the variants alone or combined showed a significant difference in their distribution between patients and controls (Table 4).

**Table 4:** Frequency data of exon 3 variants after extension of screening in alcoholic chronic pancreatitis (ACP) and idiopathic/hereditary chronic pancreatitis (ICP/HP) patients and in control subjects.

Variant	Location	Patients	Controls	*p*-Value
		**ACP**		
c.145−45G>T	Intron 2	1/446 (0.2%)	0/413	n.s
c.330C>T, (p.( = ))	Exon 3	2/446 (0.4%)	2/413 (0.5%)	n.s
c.379G>T, p.E127X	Exon 3	1/446 (0.2%)	0/413	n.s
c.473C>T, p.P158L	Exon 3	0/446	1/413 (0.2%)	n.s
c.577C>T, p.P193S	Exon 3	2/446 (0.4%)	2/413 (0.5%)	n.s
c.621C>T, (p.( = ))	Exon 3	2/446 (0.4%)	0/413	n.s
		**ICP/HP**		
c.407G>A, p.R136H	Exon 3	1/248 (0.4%)	0/413	n.s
c.577C>T, p.P193S	Exon 3	2/248 (0.8%)	2/413 (0.5%)	n.s

Abbreviations: n.s. = not significant.

## Discussion

The current study investigates the influence of genetic *HMOX1* alterations in different types of pancreatitis since there is evidence that HMOX1 induction might influence the development and the course of AP and the pathogenesis of CP by inhibition of PSC proliferation. The finding of a genetic association with different pancreatitis forms would support efforts to study HMOX1 induction by drugs or by application of hemin in a clinical setting. However, we did not find any association between the *HMOX1 GT*-repeat and any of the investigated pancreatitis phenotypes. We observed a trend towards an enrichment of S-alleles in AP and ACP, but this finding is counterintuitive as the S-allele has been associated with more HMOX1 activity and thereby probably protects against the development of pancreatitis [15].

In earlier studies, the *GT*-repeat length in the *HMOX1* promoter has been associated with several human diseases. To cite coronary artery disease patients as an example, patients with S-alleles had a higher risk at restenosis after coronary stenting [24]. Apart from the *GT*-repeat, previous studies implied a potential influence of the AA-genotype of SNP rs2071746 (g.4613A>T) located in the 5′-region of *HMOX1* in hypertension and coronary artery disease [25], [26]. On the functional level, the *A* allele and longer (>30 *GT*) repeats have been shown to elicit a higher basal promoter activity [25]. We investigated this variant in our patients and controls and found no association with any type of pancreatitis. As such, our data probably rule out that *HMOX1* promoter variants modulate the risk for pancreatitis of any aetiology. In addition, smoking represents a risk factor for pancreatitis and *HMOX1* alterations seem to influence the susceptibility to smoking related disorders [27]. However, we were not able to demonstrate differences of *HMOX1* alterations in patients that were smokers compared to non-smokers (data not shown). Noteworthy, the number of individuals in that smoking habits were available was rather small (n = 65) and as such these results have to be interpreted with caution.

In ICP/HP several genetic associations have been described with rare variants over the last years [4]–[10]. To rule out an association of rare *HMOX1* variants with diverse CP forms we extensively analysed the *HMOX1* coding regions by DNA sequencing. In summary, none of the detected non-synonymous, synonymous or non-coding variants showed an association with ICP/HP or ACP even after extension of our screening cohorts. Noteworthy, in most of the disease entities associated with *GT*-repeat variants the coding region has not been investigated comprehensively. Therefore, the role of *HMOX1* coding variants in other disease forms unlike pancreatitis is not thoroughly clarified.

### Study limitations

Our study has some limitations. The control group used for comparison are blood donors. As such this introduces potential bias because ALC, ACP or other pancreatitis forms might be present among blood donors. We surmise that the contribution of such a bias is rather small. In Germany all blood donors are routinely screened for elevated liver enzymes and persons with chronic alcohol abuse are not suitable to donate blood. Lastly, the number of ALC patients is relatively small which limits the power of this part of our study.

In conclusion, our results implicate that *HMOX1* variants are unimportant in different forms of pancreatitis and ALC. Notably, HMOX1 is induced by different stimuli and the impact of genetic variants might be minor for a substantial decrease or increase of HMOX1 expression in AP, CP or ALC. Although data obtained in experimentally induced AP picture HMOX1 as a potential target in pancreatitis, we cannot further support this view on the basis of our genetic case control study. Nevertheless, pharmacologic approaches influencing HMOX1 activity might have a benefical effect even in humans.

## Supporting Information

Table S1 **Oligonucleotide sequences of the primers used for PCR (upper section), DNA-sequencing (lower section), and their annealing temperatures in °C.** Abbreviations: PCR = polymerase chain reaction, SEQ = sequencing, F = forward, R = reverse.
(DOCX)

Table S2 **Frequencies of common intronic variants in ACP, ICP/HP, and ALC patients.** P-values are given for calculations in a dominant model (defined as (e.g. for c.144+4T>C): TT vs. TC+CC). In addition a recessive model was computed and allele frequencies were compared. Only for variant c.736+270T>C allele frequencies differed significantly between the ACP patients and controls (P-value = 0.041, OR 1.5, 95% CI 1.02–2.13). However, the p-value did not withstand Bonferroni correction (P_c_-value = 0.2). Abbreviations: het. = heterozygous, hom. = homozygous, n.s. = not significant, OR = odds ratio. * p-value given for comparison between ACP patients and controls (before Bonferroni correction).
(DOCX)
