# Supplementary material for: Genetic Analyses of Heme Oxygenase 1 (HMOX1) in Different Forms of Pancreatitis
Source: PLoS One. 2012 May 30;7(5):e37981. doi: 10.1371/journal.pone.0037981 (PMC3364204; doi:10.1371/journal.pone.0037981)
Supplement: Table S1 — Oligonucleotide sequences of the primers used for PCR (upper section), DNA-sequencing (lower section), and their annealing temperatures in °C. Abbreviations: PCR = polymerase chain reaction, SEQ = sequencing, F = forward, R = reverse. (DOCX) [file pone.0037981.s001.docx]

| **Exon (PCR)** | **F-Primer** | **R-Primer** | **Annealing** |
| --- | --- | --- | --- |
| 1 | 5’-CAGCAGGGAAGCAGTTTGTATG-3’ | 5’-CCCAGTTCTGACACCAGCTAAC-3’ | 62°C |
| 2 | 5’-GAGGATGGGAGTCTCTTGAAGG-3’ | 5’-AGAGGGTGCAGATTGCAAAAG-3’ | 63°C |
| 3 | 5’-GCTGCTGTGTGAAGAGGATTGT-3’ | 5’-GAATTCATCCTTCCAGGCTTTG-3’ | 63°C |
| 4 | 5’-CCGGCCAATATTTTTCTTACCA-3’ | 5’-GCCTGCACTCCACTTCTCATAC-3’ | 63°C |
| 5 | 5’-TTAAGGAGAGGACAGGGAGCAG-3’ | 5’-AGTTAGACCAAGGCCACAGTGC-3’ | 64°C |
| **Exon (SEQ)** |  |  |  |
| 1 | 5’-GTCCTATGGCCAGACTTTGT-3’ |  | 63°C |
| 2 |  | 5’-GGTTGATTTCAGCCTTTTCCAC-3’ | 63°C |
| 3 | 5’-GCTGTGTGAAGAGGATTGTAGC-3’ |  | 63°C |
| 4 | 5’-GCCAATATTTTTCTTACCATCT-3’ |  | 63°C |
| 5 | 5’-CTGCACAGGGAAGAACAGACAG-3’ |  | 63°C |

**Table S1:** Oligonucleotide sequences of the primers used for PCR (upper section), DNA-sequencing (lower section), and their annealing temperatures in °C. Abbreviations: PCR=polymerase chain reaction, SEQ=sequencing, F=forward, R=reverse.
