# Supplementary material for: Genetic Analyses of Heme Oxygenase 1 (HMOX1) in Different Forms of Pancreatitis
Source: PLoS One. 2012 May 30;7(5):e37981. doi: 10.1371/journal.pone.0037981 (PMC3364204; doi:10.1371/journal.pone.0037981)
Supplement: Table S2 — Frequencies of common intronic variants in ACP, ICP/HP, and ALC patients. P-values are given for calculations in a dominant model (defined as (e.g. for c.144+4T>C): TT vs. TC+CC). In addition a recessive model was computed and allele frequencies were compared. Only for variant c.736+270T>C allele frequencies differed significantly between the ACP patients and controls (P-value = 0.041, OR 1.5, 95% CI 1.02–2.13). However, the p-value did not withstand Bonferroni correction (Pc-value = 0.2). Abbreviations: het. = heterozygous, hom. = homozygous, n.s. = not significant, OR = odds ratio. * p-value given for comparison between ACP patients and controls (before Bonferroni correction). (DOCX) [file pone.0037981.s002.docx]

| **Variant** | **Intron** | **Genotype** | **ACP** | **ICP/HP** | **ALC** | **Controls** | **p-Value** | **OR** |
| --- | --- | --- | --- | --- | --- | --- | --- | --- |
| c.144+4T>C rs17885925 | 1 | TT | 133/145 (91.7%) | 127/137 (92.7%) | 128/146 (87.7%) | 133/150 (88.7%) | 0.04* | 1.5* |
|  |  | TC | 12/145 (8.3%) | 10/137 (7.3%) | 17/146 (11.6%) | 17/150 (11.3%) |  |  |
|  |  | CC | 0/145 | 0/137 | 1/146 (0.7%) | 0/150 |  |  |
| c.145-19C>T rs17879606 | 1 | CC | 134/145 (92.4%) | 127/138 (92%) | 128/147 (87.1%) | 133/151 (88.1%) | n.s. | - |
|  |  | CT | 11/145 (7.6%) | 11/138 (8%) | 18/147 (12.2%) | 18/151 (11.9%) |  |  |
|  |  | TT | 0/145 | 0/138 | 1/147 (0.7%) | 0/151 |  |  |
| c.736+52delTins  TGGCTGTCTGACT  rs17882597 | 4 | WT | 131/145 (90.3%) | 127/138 (92%) | 136/147 (92.5%) | 135/151 (89.4%) | n.s. | - |
|  |  | het. | 14/145 (9.7%) | 11/138 (8%) | 11/147 (7.5%) | 16/151 (10.6%) |  |  |
|  |  | hom. | 0/145 | 0/138 | 0/147 | 0/151 |  |  |
| c.736+226A>G  rs2269533 | 4 | AA | 47/130 (36.2%) | 57/127 (44.9%) | 48/136 (35.3%) | 58/135 (43%) | n.s. | - |
|  |  | AG | 62/130 (47.7%) | 54/127 (42.5%) | 63/136 (46.3%) | 61/135 (45.2%) |  |  |
|  |  | GG | 21/130 (16.2%) | 16/127 (12.6%) | 25/136 (18.4%) | 16/135 (11.9%) |  |  |
| c.736+270T>C  rs2269534 | 4 | TT | 56/130 (43.1%) | 67/127 (52.8%) | 57/136 (41.9%) | 71/135 (52.6%) | n.s. | - |
|  |  | TC | 54/130 (41.5%) | 46/127 (36.2%) | 67/136 (49.3%) | 53/135 (39.3%) |  |  |
|  |  | CC | 20/130 (15.4%) | 14/127 (11%) | 12/136 (8.8%) | 11/135 (8.1%) |  |  |

**Table S2:** Frequencies of common intronic variants in ACP, ICP/HP, and ALC patients. P-values are given for calculations in a dominant model (defined as (e.g. for c.144+4T>C): TT vs. TC+CC). In addition a recessive model was computed and allele frequencies were compared. Only for variant c.736+270T>C allele frequencies differed significantly between the ACP patients and controls (P-value=0.041, OR 1.5, 95% CI 1.02-2.13). However, the p-value did not withstand Bonferroni correction (P_c_-value=0.2). Abbreviations: het.=heterozygous, hom.=homozygous, n.s.=not significant, OR=odds ratio. * p-value given for comparison between ACP patients and controls (before Bonferroni correction).
